# Surface Display of Peptides Corresponding to the Heptad Repeat 2 Domain of the Feline Enteric Coronavirus Spike Protein on *Bacillus subtilis* Spores Elicits Protective Immune Responses Against Homologous Infection in a Feline Aminopeptidase-N-Transduced Mouse Model

**DOI:** 10.3389/fimmu.2022.925922

**Published:** 2022-06-28

**Authors:** Chu Chen, Ya-Li Li, Fang-Li Lv, Ling-Dong Xu, Yao-Wei Huang

**Affiliations:** ^1^ Department of Veterinary Medicine, Zhejiang University, Hangzhou, China; ^2^ Guangdong Laboratory for Lingnan Modern Agriculture, College of Veterinary Medicine, South China Agricultural University, Guangzhou, China

**Keywords:** feline coronavirus (FCoV), heptad repeat 2 (HR2), *Bacillus subtilis*, mouse model, recombinant oral vaccine

## Abstract

Although feline coronavirus (FCoV) infection is extremely common in cats, there are currently few effective treatments. A peptide derived from the heptad repeat 2 (HR2) domain of the coronavirus (CoV) spike protein has shown effective for inhibition of various human and animal CoVs *in vitro*, but further use of FCoV-HR2 *in vivo* has been limited by lack of practical delivery vectors and small animal infection model. To overcome these technical challenges, we first constructed a recombinant *Bacillus subtilis* (rBS^CotB-HR2P^) expressing spore coat protein B (CotB) fused to an HR2-derived peptide (HR2P) from a serotype II feline enteric CoV (FECV). Immunogenic capacity was evaluated in mice after intragastric or intranasal administration, showing that recombinant spores could trigger strong specific cellular and humoral immune responses. Furthermore, we developed a novel mouse model for FECV infection by transduction with its primary receptor (feline aminopeptidase N) using an E1/E3-deleted adenovirus type 5 vector. This model can be used to study the antiviral immune response and evaluate vaccines or drugs, and is an applicable choice to replace cats for the study of FECV. Oral administration of rBS^CotB-HR2P^ in this mouse model effectively protected against FECV challenge and significantly reduced pathology in the digestive tract. Owing to its safety, low cost, and probiotic features, rBS^CotB-HR2P^ is a promising oral vaccine candidate for use against FECV/FCoV infection in cats.

## Introduction

Feline coronavirus (FCoV) is an important gastrointestinal pathogen of domestic cats and is known to be prevalent in catteries and multiple-cat households. FCoV belongs to the genus *Alphacoronavirus*, which contains the closely related canine CoVs and porcine transmissible gastroenteritis virus (TGEV) (1–3). Feline enteric CoV (FECV) and related mutant feline infectious peritonitis virus (FIPV) coexist in the environment and are indistinguishable in antigen, serology, and morphology (4). Although the vast majority of FECVs are benign and cause only mild diarrhea, FIPV can lead to a lethal disease (5), with no effective vaccine having been developed to date (6–8). There are two serotypes of FCoV (9); some studies have shown that serotype II FCoV uses feline aminopeptidase N (fAPN) as a receptor for invading cells, whereas the receptors for type I FCoV remain unclear (9, 10). Although some encouraging progress has been made with potential anti-FCoV therapeutic agents such as GS-441524 and GC376, prevention, and treatment of FCoV infection still face considerable challenges (11).

The CoV S glycoprotein (composed of S1 and S2 subunits) is mainly responsible for binding to the cell surface receptor (12–15). S1 binds to the host receptor and causes a conformational change in S2, which exposes it to the target cell membrane, and the highly conserved heptad repeat regions (HR1 and HR2) interact to form a stable six-helix structure that plays an important role in viral fusion (16). Exogenous soluble HR2 peptides can bind to viral HR1, thereby efficiently blocking viral entry into the cell (17, 18). HRs of severe acute respiratory syndrome (SARS)-CoV, novel SARS-CoV-2, Middle East respiratory syndrome (MERS)-CoV, and porcine epidemic diarrhea virus (PEDV) can all inhibit viral invasion (17–20). As a typical class I enveloped virus, FCoV is expected to use a similar membrane fusion mechanism for viral entry, and the HR2 of FCoV has also been proven to be effective in inhibiting viral invasion (21, 22). Additionally, our previous study demonstrated that the HR2 of PEDV contains a possible neutralizing epitope with high immunogenicity, and polyclonal antiserum against this domain has a high neutralization activity (18). Thus, we consider HR2 a good candidate antigen to base further vaccine development on. However, the application of HR2, especially for animals, is very challenging due to its poor stability and short half-life (23, 24).

Since the main target of FCoV infection is the intestinal tract, an efficient vaccine must elicit a strong mucosal immune response to protect the body from infection (25, 26). *Bacillus subtilis* is a natural probiotic that can induce a strong mucosal immune response through various immune pathways, promoting the expression of cytokines and secretory immunoglobulin A (sIgA) (27). Additionally, *B. subtilis* can adhere to intestinal epithelial cells and accumulate in the digestive tract. As a natural adjuvant, it is widely used in vaccine preparations (27–29), and it can protect exogenous antigens from being degraded by the digestive tract before they pass through the gastrointestinal barrier (30). The *B. subtilis* spore surface display (BSSD) technique is considered one of the most promising methods for expressing heterologous proteins with high activity and stability (31, 32).

In this study, we designed and developed a novel FCoV vaccine candidate based on BSSD of recombinant HR2 peptide (HR2P) and evaluated its immunogenicity and protection in a new type II FCoV infection model by fAPN transduction in mice.

## Materials and Methods

### Cells, Virus, and Animals

CRFK (feline kidney) cells were cultured in Dulbecco’s modified Eagle’s medium (DMEM, Gibco) supplemented with 10% fetal bovine serum (FBS, Gibco) and 1% penicillin/streptomycin (w/v) in a humidified 5% CO_2_ incubator at 37°C. The type II FECV 79-1683 strain (33) was a gift from Professor Rong Ye (Shanghai Medical College of Fudan University). The virus titer of FECV was determined by endpoint dilutions as 50% tissue culture infective dose (TCID_50_) on CRFK. Virus stocks were stored at −80°C until use. Six-week-old female BALB/c mice were purchased from the Zhejiang Academy of Medical Science (Hangzhou, China) and raised in a sterilized room with the temperature set at 25 to 27°C, with a 12 h daily light cycle, and fed sterilized food and water.

### Design and Expression of HR2 Peptides in *Escherichia coli*

**Figure 1:**
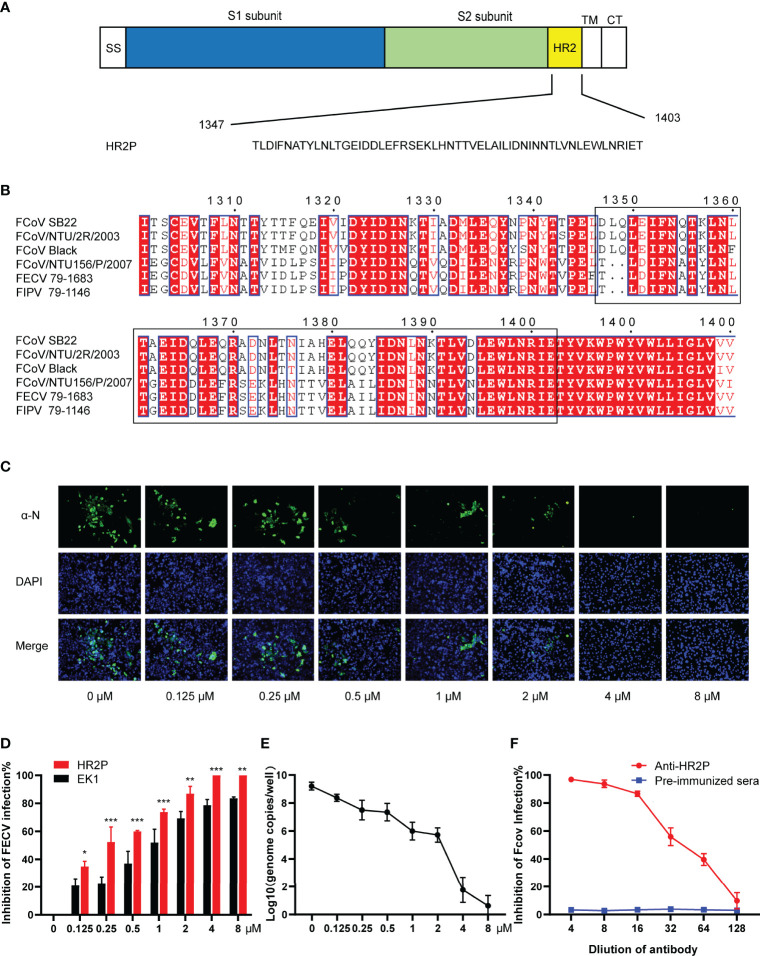
The FECV spike HR2 peptide (HR2P) exhibits antiviral activity and elicits neutralizing antibodies. **(A)** Schematic of the S glycoprotein, showing heptad repeat 2 (HR2) located within the S2 subunit. Other domains shown include SS (signal sequence), TM (transmembrane domain), and CT (cytoplasmic tail). The HR2 sequence was predicted by the LearnCoil-VMF program. **(B)** Multiple sequence alignment of HR2 regions (1347-1403 aa based on the FECV strain 79-1683) from type I (FCoV-SB22, GenBank accession no. MH817484; FCoV/NTU/2R/2003, DQ160294; FCoV Black, EU186072) and type II (FCoV/NTU156/P/2007, GQ152141.1; FECV 79-1683, X80799.1; FIPV 79-1146, DQ010921.1) strains by using ClustalX1.8 and ESPript3.1 program. Residues highlighted in red are completely conserved. **(C)** Inhibition of FECV infection by HR2P *in vitro*. FECV (MOI = 0.01) was preincubated with soluble HR2P-His at various concentrations (0.125, 0.25, 0.5, 1, 2, 4, and 8 μM) for 1 h at 37°C, and then used to infect CRFK cells. Viral infection was determined at 24 hpi by IFA, using a polyclonal antibody against the viral N protein, followed by incubation with a FITC-conjugated secondary antibody. **(D)** Comparison of peptide (HR2P and EK1) inhibition of FECV infection. Figure represents mean ± standard error of triplicate wells; *P <.05; **P <.01 ; ***P <005. **(E)** Infection of FECV in CRFK cells was inhibited by HR2P-His at various concentrations (0.125, 0.25, 0.5, 1, 2, 4, and 8 μM) within 24 h. FECV RNA titers were measured by qRT-PCR targeting the 3′-UTR. *P <.05; **P <.01 ; ***P <005. **(F)** Neutralizing activity of polyclonal antiserum raised against HR2P. FECV (100 TCID_50_/well) was preincubated with different dilutions of antiserum and used to infect CRFK cells, and the reduction in viral titer was quantified. The figure represents mean values from three independent experiments, and the error bars indicate the standard deviation. The figure represents the mean ± standard error of triplicate wells; *P <.05; **P <.01 ; ***P <005.

The cloning strategy for the HR2 region of the FECV S protein was designed based on the type II Felix isolate, predicted using the computer software LearnCoil-VMF (http://night-ingale.lcs.mit.edu/cgi-bin/vmf), with a peptide sequence as shown in **Figure 1A**. Gene fragments corresponding to the desired peptide were amplified through polymerase chain reaction (PCR) on a full-length FECV S construct, and the product was inserted into a pET32a vector (containing a His tag) after restriction endonuclease digestion with *BamH*I and *Xho*I. The prokaryotic expression vector pET32a-HR2P was constructed and transformed into BL21 Chemically Competent Cells (TransGen, China), and a single colony was inoculated into Luria–Bertani (LB) broth containing 50 mg/L ampicillin (Sigma, USA) and incubated at 37°C. Overnight cultures were transferred into 100 ml of fresh LB medium for large-scale protein production at 37°C. Expression of recombinant HR2P-His was induced with isopropyl-β-D-thiogalactoside (IPTG) (Sigma, USA) at a final concentration of 1 mM when Abs_600 nm_ reached 0.5. After induction for 5 h at 37°C, cells were harvested by centrifugation at 10,000×*g* for 15 min at 4°C. The bacterial cells were disrupted by ultrasonication, and lysates were added to His Pur Ni-NTA columns (TransGen, Beijing, China) and filtered by gravity flow. Purified HR2P-His was separated on a 12% SDS-PAGE gel, transferred to a PVDF membrane and blocked with nonfat milk for 1 h at room temperature. After three washes with Tris-buffered saline Tween-20 (TBST), the membranes were incubated with mouse anti-His diluted with TBST in 5% bovine serum albumin (BSA) at 4°C overnight. Following three more washes with TBST, the membrane was incubated with a horseradish peroxidase (HRP)-conjugated goat anti-mouse IgG for 1 h at 37°C. Blots were visualized using an ECL Western Blot detection kit (FUDE, China) as specified by the manufacturer. The protein concentration was measured using the enhanced BCA protein assay kit (Beyotime, China) according to the instructions of the manufacturer.

### Cytotoxicity Assay and Virus Inhibitory Effect of the Recombinant HR2 Peptide

To determine the cytotoxicity of HR2P, CRFK cells (3 ×10^4^ cells/ml) were grown in 96-well plates for 24 h, then pre-incubated with dilutions (0.5, 1, 2, 4, 8, and 16 μM) of HR2P in triplicate at 37°C for 72 h and assessed with the Cell Counting Kit-8 (Beyotime, China). After addition of 10 μl of CCK-8 solution to each well, plates were incubated for 1–4 h at 37°C, and Abs_450 nm_ was measured with a microplate reader. Cell viability was calculated as the relative absorbance at 450 nm of samples treated with HR2P relative to that of untreated cells.

CRFK cells were seeded at 80% confluency in 48-well plates and infected 24 h later with FECV [multiplicity of infection (MOI) = 0.01] mixed with HR2P at a range of concentrations (0.5, 1, 2, 4, 8, and 16 μM) for 2 h at 37°C. Negative control cultures were also treated with soluble HR2P diluted in maintenance media (MM) containing DMEM at the same concentrations. An indirect immunofluorescence assay (IFA) was used to detect infected cells at 24 h, and the inhibition rate was calculated by fluorescence area analysis. Inhibitory concentration 50% (IC_50_) values were calculated as previously described (18). To determine the virus content in cells infected with different concentrations of HR2P-FECV mixture, CRFK cells (4 × 10^5^ cells/ml) were grown in 24-well plates for 24 h. Total RNA was extracted from supernatant medium and cell lysates of HR2P-FECV inoculated cells using trizol (TaKaRa, China). The FECV RNA titer was monitored by one-step quantitative reverse transcription PCR (RT-qPCR) targeting the 3’-UTR with the primers 5’-AAGCACGTGTAATGGGAGGT-3’ and 5’-CACTAGATCCAGACGTTAGC-3’ and the probe FAM-TCCGCTATGACGAGCCAACAATGGA. Standard curves were performed to allow absolute quantitation of FECV RNA copy numbers based on the levels of *in vitro*-transcribed RNA containing the targeting sequences. Results are expressed as the average of triplicates ± standard deviation, and all experiments were conducted in parallel with His fusion peptide and the His control in different treatment.

### Preparation of Anti-HR2P Polyclonal Antibody and Virus Neutralization Experiment

Mice were injected intramuscularly (IM) with purified soluble HR2P (50 μg per mouse) and boosted with 100 μg per mouse at 14, 24, and 34 d post-inoculation. Serum was collected and HR2P was detected by ELISA 7 days after the final booster. Serial dilutions of 50 μl pre-immune serum, anti-HR2P antiserum were mixed with FECV (MOI = 0.01) in MM, and cultured at 37°C for 1 h to form virus–antibody complexes. The mixture was added to CRFK cells grown in 48-well culture plates with DMEM containing 10% FBS, and after 1 h of culture at 37°C, viruses were removed by three washes in PBS (phosphate buffered saline). At 48 h post-infection, the cells were fixed with 4% paraformaldehyde and positive cells were counted after IFA.

### Surface Display of HR2 Peptide on *B. subtilis* Spores

**Figure 2:**
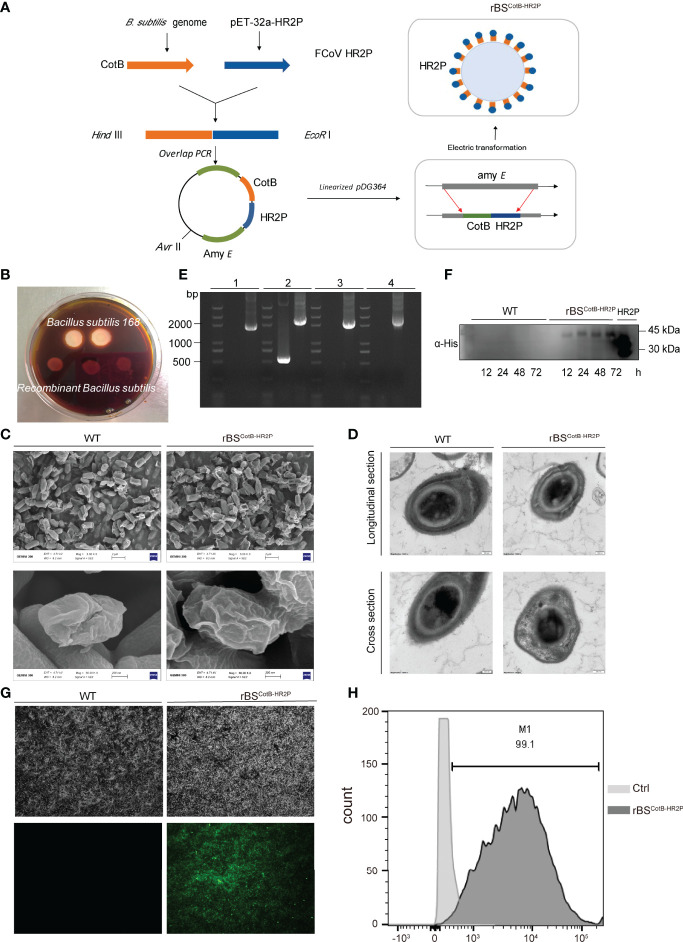
Construction of recombinant *B. subtilis* with surface display of FECV HR2P. **(A)** Genetic engineering of a recombinant *B. subtilis* spore with CotB-HR2P on its surface (rBS^CoTB-HR2P^). **(B)** Starch hydrolysis test. *B. subtilis* 168 and rBS^CoTB-HR2P^ were cultivated on a medium containing 1% starch and stained with iodine. **(C)** Images of WT and RBS^CotB-HR2P^ spores obtained by scanning electron microscopy; scale bars: 2 μm (upper); 200 nm (lower). **(D)** Images of WT and rBS^CotB-HR2P^ spores were obtained by transmission electron microscopy; scale bars: 200 nm. **(E)** PCR analysis of *B. subtilis* using different primer pairs: (1) CotB-F and HR2P-R; (2) amyE-F and amyE-R; (3) amyE-F and HR2P-R; and (4) CotB-F and amyE-R. **(F)** Western blot detection of the CotB-HR2P fusion protein from wild-type (WT) and rBS^CotB-HR2P^ spores induced by DSM at different time points (12, 24, 48, and 72 h), probed with polyclonal mouse anti-His antibody. **(G)** Immunofluorescence of CotB-HR2P on the spore surface at 72 h after induction of wild-type (WT) and RBS^CotB-HR2P^. **(H)** Flow cytometry analysis of CotB-HR2P fusion protein expressed on the surfaces of WT and rBS^CotB-HR2P^ spores.

The same FECV HR2 coding sequence described above was inserted into the *B. subtilis* genome through homologous recombination to produce a bacterial strain expressing HR2P on its surface (**Figure 2**). First, the genomic DNA of *B. subtilis* strain 168 (34) was extracted as a template to amplify the CotB gene (1,088 bp). The HR2 sequence was amplified using pET32a-HR2P as a template, and overlap extension PCR was used to join it with the CotB amplicon. CotB-HR2P was digested with *Hind*III and *EcoR*I (Takara, China) and inserted into a similarly digested pDG364 vector, subcloned and sequenced to confirm the lack of unwanted mutation (SunYa, China). The resulting pDG364-CotB-HR2P was linearized by single enzyme digestion (AvrII) (TaKaRa, China) and transformed into the amylase E gene of the competent *B. subtilis* genome by electroporation. Chromosomal DNA was extracted from rBS^CotB-HR2P^ and amylase-inactivated strains and identified by PCR with different primer sets (CotB-F: 5’-CCCAAGCTTAGCAAGAGGAGAATGAAATATCATTCAAATAATGAAATATC-3’; CotB-R: 5’-ATAGACTATCACTGGAAACGTAAATTT-3’; HR2P-F: 5’-ATCACTGGAAACGTAAATTTATGAGCGATAAAATTATTCACCTGA-3’; HR2P-R: 5’-CCGGAATTCTCATGTGGTTTCAATACGATTTAACCA-3’; AmyE-F: CCAATGAGGTTAAGAGTATTCC-3’; AmyE-R: 5’-CGAGAAGCTATCACCGCCCAGC-3’). The recombinant *B. subtilis* was induced and cultured at 37°C for 72 h using Difco Sporulation Medium (DSM). Bacterial spores were purified by 4 mg/ml lysozyme and washed with 1 M NaCl, 1 M KCl, and 1 M benzyl sulfonyl fluoride solution. After being incubated at 68°C for 1 h, pure spores were obtained, resuspended in PBS, and stored at −80°C.

### Western Blot for Detection of HR2 Peptide Expression in *B. subtilis* Spores

Collected spores were incubated with SDS-DTT (0.5% SDS, dithiothreitol 0.1%, 0.1 M NaCl) at 37°C for 30 min, and run on 12% SDS-PAGE followed by Coomassie brilliant blue staining, and then transferred to polyvinylidene fluoride filters (Sigma, USA). Filters were sealed overnight at 4°C in 5% skim milk in PBST (containing 0.05% (v/v) Tween 20), and then probed with primary anti-His antibody (1:1,000 in PBST) and secondary antibody against HRP-conjugated goat anti-rabbit IgG (1:5,000 in PBST). The signal was detected by Pico ECL (China, FUDE).

### Immunofluorescence and Flow Cytometry

For the detection of HR2P on the surface of spores, 1 ml of purified spore suspension harvested after 72 h of *B. subtilis* growth was added to a glass slide, and after drying, 5% BSA was added for 2 h. Anti-His antibody (1:1,000 in PBST) was added and incubated at 4°C overnight, followed by goat anti-rabbit IgG (Invitrogen; 1:500 in PBST) as secondary antibody. Spores were visualized after the addition of fluorescein isothiocyanate (FITC) on a fluorescence microscope (Leica, Germany) equipped with an Olympus camera (miniature DP72; Olympus, Japan).

For flow cytometry experiments, *B. subtilis* cultures were collected after 72 h of induction, centrifuged at 4,000×*g*, and the medium was discarded, and cell pellets were resuspended in PBS. After repeatedly washing with PBS, bacterial suspensions were incubated overnight with anti-His antibody at 4°C, washed 3 times in PBS, and incubated with goat anti-rabbit IgG (1:1,000 in PBST) and FITC. After three washings, spores were finally re-suspended in 1 ml of PBS, and at least 10^4^ spores were detected using an FC500MPL flow cytometer (FACSVerse). Expression of the CotB-HR2P fusion protein was analyzed by FlowJo software (TreeStar).

### Observation of Spore Structure

Purified wild-type (WT) and recombinant rBS^CotB-HR2P^ spores were collected and immobilized overnight in 3% glutaraldehyde at 4°C, and then serially dehydrated in graded ethanol (50, 70, 90, and 100%). After subsequent critical point drying and sputter coating, the samples were processed and photographed on an SU-70 scanning electron microscope (Hitachi, Japan). Ultra-thin slices were mounted on 230-mesh copper mesh, stained with 1% uranyl acetate lead citrate, and the spores were observed and photographed on an H-9500 transmission electron microscope (Hitachi, Japan).

### Ethics Statement

Animal use was approved by the Zhejiang University Experimental Animals Ethics Committee (IACUC approval no. ZJU20181049). All animals were handled following guidelines for the care and use of laboratory animals set by the same committee.

### Immunogenicity Study of Recombinant *B. subtilis*

Groups of six five-week-old female mice were inoculated with 10^8^ CFU rBS^CotB-HR2P^ spores or *B. subtilis* spores by nasal drop or gavage. A positive control group received 100 μg of purified HR2P by IM injection and was boosted with the same dose at 14 days post-vaccination (dpv), whereas PBS was given by gavage to a negative control group. All six groups were immunized on days 1–3, 8–10, and 15–17 as described previously (35). Specific anti-HR2P antibodies were monitored in serum once a week after the first immunization, and the level of anti-HR2P sIgA in the intestine was detected after euthanasia in the fifth week. After isolating splenic lymphocytes from mice in the fifth week, they were stimulated with LPS (5 mg/ml; Sigma, Germany) or purified HR2P (15 mg/ml) as described previously (36). The cells were evaluated for proliferation using a CCK8 assay kit (Sangon Biotech, China) according to the instructions of the manufacturer.

### Determination of Antibody Levels by ELISA

Anti-HR2P IgG and sIgA were determined by ELISA, using plates (Bethy) coated with 50 μl of purified HR2P dissolved in coating buffer (0.05 M carbonate-bicarbonate [pH 9.6]) at a concentration of 1,000 ng/ml. Plates were blocked with 5% skim milk in coating buffer for 18 h at room temperature, washed three times in PBST, and incubated at 37°C for 2 h in 1:400 serum or intestinal mucus in PBST. Subsequently, HRP-conjugated goat anti-mouse IgG (1:5,000; Abcam, UK), and goat anti-mouse IgA (1:5,000; Abcam) were used as suitable secondary antibodies. After 1 h of incubation, plates were washed again, and 100 μl of substrate TMB (3,3’,5,5’-tetramethylbenzidine; BD Biosciences) was added. The reaction was stopped after 5 min of incubation in the dark by adding 50 ml of 2 M H_2_SO_4_, and the plates were read three times at 450 nm in a microplate ELISA reader (Bio-Rad, Japan). Negative-control wells incubated with naïve sera were included in each plate. The results are expressed as the average of three OD_450_ values. Sera collected from mice at the third week post-inoculation were co-incubated with FECV for viral neutralization assays (1:4 to 1:128 dilutions) as described above.

### Detection of Bacterial Colonization of the Small Intestine

Small intestinal segments were collected from mice sacrificed in the fifth week post-inoculation, rinsed 3 times with PBS, and the intestinal contents were collected. After centrifugation at 700×*g* for 5 min, the supernatants were inoculated onto LB agar plates containing 20 μg/ml chloramphenicol and cultured overnight at 37°C before counting the colonies. Chromosomal DNA was extracted from the colonies on the plates and identified by PCR with different primer sets (CotB-F: 5’-CCCAAGCTTAGCAAGAGGAGAATGAAATATCATTCAAATAATGAAATATC-3’; HR2P-R: 5’-CCGGAATTCTCATGTGGTTTCAATACGATTTAACCA-3’).

### Generation of Recombinant Adenoviral Vectors Expressing fAPN (Ad5-fAPN)

Recombinant adenoviral vectors expressing fAPN (Ad5-fAPN) or green fluorescent protein (Ad5-GFP) with Flag tags were prepared and used to transduce 293 cells at an MOI of 0.01 for 4 h at 37°C as previously described (37, 38). At 48 h post-transduction, samples were collected for western blotting as described above. For confocal imaging, transduced cells were seeded in a 35-mm confocal dish in complete medium for 24 h of incubation at 37°C with 5% CO_2_. Viral growth curves were determined as described previously (39).

### Development of a FECV Infection Model and Pathogenicity Study in Ad5-fAPN-Transduced Mice

Five-week-old female mice were transduced intraperitoneally with 2.5 × 10^8^ TCID_50_ of Ad5-fAPN or Ad5-GFP (as control) in 1 ml of DMEM, with 6 mice in each group. Five days post-transduction, mice were infected intraperitoneally with FECV (10^5^ TCID_50_) in a total volume of 1 ml of DMEM. Fecal and intestinal tissue samples were continuously collected from three mice in each group at different time points and were tested for FECV genomic copies by real-time qRT-PCR at 1, 3, 5, 7, 10, and 14 days post-infection (dpi). Serum samples were collected weekly and tested for anti-FECV IgG antibodies by ELISA. Samples of brain, liver, lung, spleen, and mesenteric membrane samples were collected and tested for FECV genomic copies at 5 dpi. Gross lesions in the intestine were examined at 5 dpi. Histological examination and immunohistochemistry were conducted in intestinal sections at 5 dpi. Intestine samples from infected and control animals were dissected, fixed in 4% paraformaldehyde for 12 h at 4°C, serially dehydrated in an ethanol gradient, embedded in paraffin, and sliced into 6-μm-thick sections. The sections were subjected to histological examination after hematoxylin and eosin (H&E) staining. Immunohistochemistry (IHC) was carried out using anti-Flag antibodies and anti-TGEV-N generated previously (40), since the N proteins of TGEV and FECV are highly similar and thus anti-TGEV-N cross-reacts with FECV.

### FECV Challenge Study in Ad5-fAPN-Transduced Mice Immunized With Recombinant *B. subtilis*

Mice were immunized with rBS^CotB-HR2P^ by gavage for three consecutive weeks as previously stated, then transduced intraperitoneally with 2.5 × 10^8^ TCID_50_ of Ad5-fAPN or Ad5-GFP in 1 ml of DMEM. At 4 d post-transduction, mice were infected intraperitoneally with FECV (10^6^ TCID_50_) in a total volume of 1 ml of DMEM. Fecal and intestinal tissue samples were continuously collected from three mice in each group at different time points and were tested for FECV genomic copies by real-time qRT-PCR at 1, 3, 5, 7, 10, and 14 dpi. Histological examination and immunohistochemistry were conducted in intestinal sections at 5 dpi.

### Statistical Analysis

The data were expressed as the mean and standard deviation (mean ± SD) and analyzed by GraphPad Prism software to make graphs and perform statistical analyses, and differences among groups were analyzed by one-way ANOVA (*, *P <*0.05; **, *P <*0.01; ***, *P <*0.001; ****, *P <*0.0001).

## Results

### Peptides Derived From the FECV HR2 Exhibit Excellent Antiviral Activity *In Vitro*, and Stimulate Strongly Neutralizing Antibodies

The HR2 region of the FECV 79-1683 S protein (1347-1403 aa) is followed by the transmembrane region and the cytoplasmic tail (**Figure 1A**). We aligned the HR2 domain sequences from different representative FCoV strains, including type I (SB22, NTU/2R/2003, and Black) and type II (NTU156/P/2007, 79-1683, and 79-1146), confirming that the HR2 domain is highly conserved between different strains (**Figure 1B**). According to previous HR2-related studies on the other CoVs (17–19, 21, 22), it can be speculated that the FECV HR2 domain peptide we designed would be effective as an antiviral or vaccine antigen for many types of FCoV.

The HR2 domain with a 6x His-tag was cloned and expressed in a prokaryotic expression, vector pET32a-HR2P. After purification of the recombinant soluble product HR2P, we tested the cytotoxicity of the peptide in CRFK cells, with monolayers exposed to a maximum concentration of 8 μM of HR2P for 24 h. There was no statistically significant difference between the viability of the untreated cells (control) and the toxicity observed in cells exposed to HR2P, with greater than 95% survival in all cells as determined by CCK8 (data not shown).

Based on the cell viability results, we chose to test the *in vitro* antiviral effects of HR2P administration at 0.125, 0.25, 0.5, 1, 2, 4, and 8 μM. CRFK cells were infected with a preincubated mixture of FECV and HR2P at various concentrations. After 24 h, IFA revealed a significant reduction in viral N protein expression in infected, HR2P-treated cells that was dose-dependent (**Figure 1C**). The IC_50_ value for HR2P in this experiment was calculated to be 0.31 μM. A pan-CoV-fusion inhibitory peptide EK1 targeting the HR1 domain of the spike was used as the control (41). Compared to HR2P, EK1 was less effective against FECV, with an IC_50_ of 0.84 μM (**Figure 1D**). To quantitatively analyze the viral content in cells infected with different concentrations of the HR2P–FECV mixture, the viral RNA levels in the supernatant of the cell lysates at 24 hpi were assessed by qRT-PCR. Consistent with the IFA results, HR2P effectively inhibited FECV infection (**Figure 1E**).

Next, we wanted to test the ability of HR2P to elicit neutralizing antibodies in mice. After immunizing mice with soluble HR2P-His protein expressed in *E. coli*, we collected antiserum and tested whether it could block FECV infection *in vitro*. Whereas naïve mouse serum had no effect, serum from HR2P-immunized mice effectively dose-dependently inhibited FECV infection (**Figure 1F**). FECV infection was reduced by 50% between the 1:64 and 1:32 dilutions of serum tested, suggesting that HR2P was strongly immunogenic.

### Construction and Identification of Recombinant *B. subtilis* Spores With Surface Display of HR2P

Having validated FECV HR2P function in antiviral and eliciting neutralizing activity, as BSSD is an ideal method for antigen delivery in mice, we next constructed a recombinant *B. subtilis* strain expressing HR2P on the surface of its spores (rBS^CotB-HR2P^). Briefly, a fusion gene consisting of CotB and HR2 coding sequences was inserted into *B. subtilis via* homologous double-crossover recombination, disrupting the amylase gene (amyE) (**Figure 2A**). Thus, rBS^CotB-HR2P^ could not hydrolyze starch because of damage to the amylase gene, unlike the wildtype (**Figure 2B**). To investigate whether the insertion of an exogenous peptide affected the germination and morphology of the spores, we detected the spores by scanning electron microscopy and transmission electron microscopy. There was no change in the surface (**Figure 2C**) or internal structural morphology of *B. subtilis* spores (**Figure 2D**). To confirm the correct orientation of CotB-HR2P, PCR was conducted using multiple primer sets, with the expected size bands (1,815-, 2,371-, 2,042-, and 2,144-bp; lanes “rBS,” left to right) obtained after gel electrophoresis (**Figure 2E**). Bacterial spores of rBS^CotB-HR2P^ were induced in DSM for 72 h, with wild-type *B. subtilis* spores produced as controls. Western blotting showed CotB-HR2P in the spores from 24 to 72 h of cultivation (**Figure 2F**). Immunofluorescence confirmed surface expression of the recombinant fusion protein, with strong fluorescence observed on rBS^CotB-HR2P^ spores 72 h after induction, whereas the wildtype spores remained negative (**Figure 2G**). Flow cytometry analysis further confirmed that 75% of the recombinant spores expressed CotB-HR2P on their surface at 72 h post-induction (**Figure 2H**).

### rBS^CotB-HR2P^ Spores are Immunogenic in Mice After Oral or Nasal Administration

**Figure 3:**
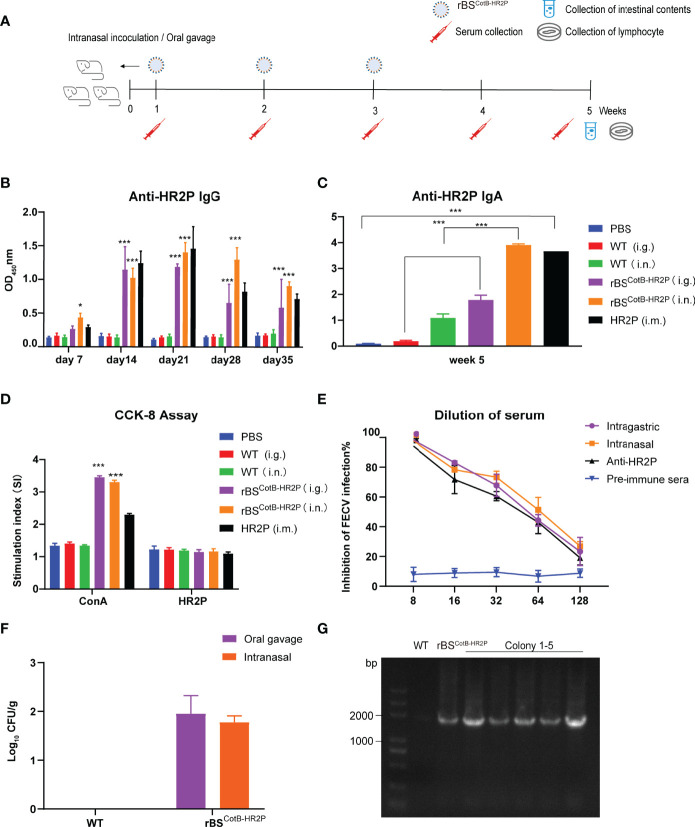
Recombinant **(B)** *subtilis* spores expressing the CotB-HR2P fusion protein induce specific humoral and cellular immune responses in mice. **(A)** Scheme of mouse experiment in which five-week-old female mice (n = 6 each group) were inoculated with either PBS (Ctrl), purified 50 μg HR2P-His peptide intramuscularly (IM), or 10^8^ CFU of the following: wildtype *B. subtilis* spores intragastric (WT i.g.); WT intranasal (i.n.); recombinant *B. subtilis* spores displaying the CotB-HR2P fusion protein (rBS^CotB-HR2P^) i.g., or rBS^CotB-HR2P^ i.n. Serum and intestinal content were collected at the indicated time points. **(B)** Anti-HR2P IgG levels in the sera of mice (n = 6 in each group) were measured weekly by ELISA. **(C)** Anti-HR2P sIgA levels in the intestinal contents of mice (n = 6 in each group) were measured at 5 weeks post-inoculation. **(D)** Proliferation of mouse splenic lymphocytes was measured by the CCK-8 assay at week 5. **(E)** Neutralization of anti-HR2P antibody in serum of mice inoculated with WT or rBS^CotB-HR2P^. CRFK cells were infected with FECV (100 TCID_50_/well) after preincubation with diluted antiserum, and the reduction in viral titer was quantified. The figure represents mean values from three independent experiments, and the error bars indicate the standard deviation. **(F)** Colonization of rBS^CotB-HR2P^ in the small intestine of mice five weeks after inoculation. **(G)** PCR analysis of bacterial colony cultured from small intestinal contents using CotB-F and HR2P-R primers. *P <.05; ***P <.005. All data are presented as means ± the SEM. Three technical replicates from a single experiment were used.

To test whether the recombinant *B. subtilis* spores produced an immune response, 5-week-old female mice were immunized with wildtype and rBS^CotB-HR2P^ spores, respectively, by oral gavage or nasal drops (**Figure 3A**). Additionally, one group was injected with IM with purified HR2P to serve as a positive control, whereas the negative control group was given PBS. Both intranasal and intragastric inoculation of rBS^CotB-HR2P^ spores induced high IgG levels in mice, peaking at week 3 and comparable to IgG levels produced by IM HR2P injection, and significantly higher than those in the negative group (*P* <.01) (**Figure 3B**). Significant levels of mucosal sIgA levels were detected in the intestinal content of rBS^CotB-HR2P^-inoculated mice at 5 weeks post-inoculation compared with the negative control (*P* <.01), and nasal drops seemed to induce higher sIgA levels (**Figure 3C**). Lymphocytes extracted from mouse spleens at 5 weeks post-inoculation were stimulated with concanavalin A (ConA) and purified HR2P to measure specific cell-mediated immune responses. The rBS^CotB-HR2P^-treated mice (both oral gavage and nasal drops) had higher levels of lymphocyte proliferation than in WT and PBS-treated mice after ConA stimulation (*P* <.01) (**Figure 3D**). Subsequently, the collected mouse serum was tested for FECV neutralizing antibodies (**Figure 3E**). Antiserum from rBS^CotB-HR2P^-inoculated mice suppressed 50% of FECV infection between 1:32 and 1:64 dilution, whereas wildtype *B. subtilis*-inoculated mice did not develop neutralizing antibodies. The mice inoculated with rBS^CotB-HR2P^ appeared to produce slightly higher levels of neutralizing antibodies than the group given HR2P IM. All the data suggested that the engineered rBS^CotB-HR2P^ had good immunogenicity.

To evaluate colonization of recombinant *B. subtilis* in the inoculated mice, small intestinal content was collected at week 5, diluted and cultured at 37°C for 17–24 h. The results of colony counts are shown in **Figure 3F**. The number of bacteria colonizing the gavage group was slightly higher than that of the nasally inoculated group, but recombinant *B. subtilis* could to colonize the intestinal tract of the mice for at least five weeks regardless of inoculation route. We extracted genomic DNA from colonies on the plate and amplified CotB-HR2P, producing bands of a corresponding size of 1,815 bp in **Figure 3G** (WT, recombinant *B. subtilis*, colonies 1–5, left to right).

### Development of Mice Susceptible to Type II FECV Infection

**Figure 4:**
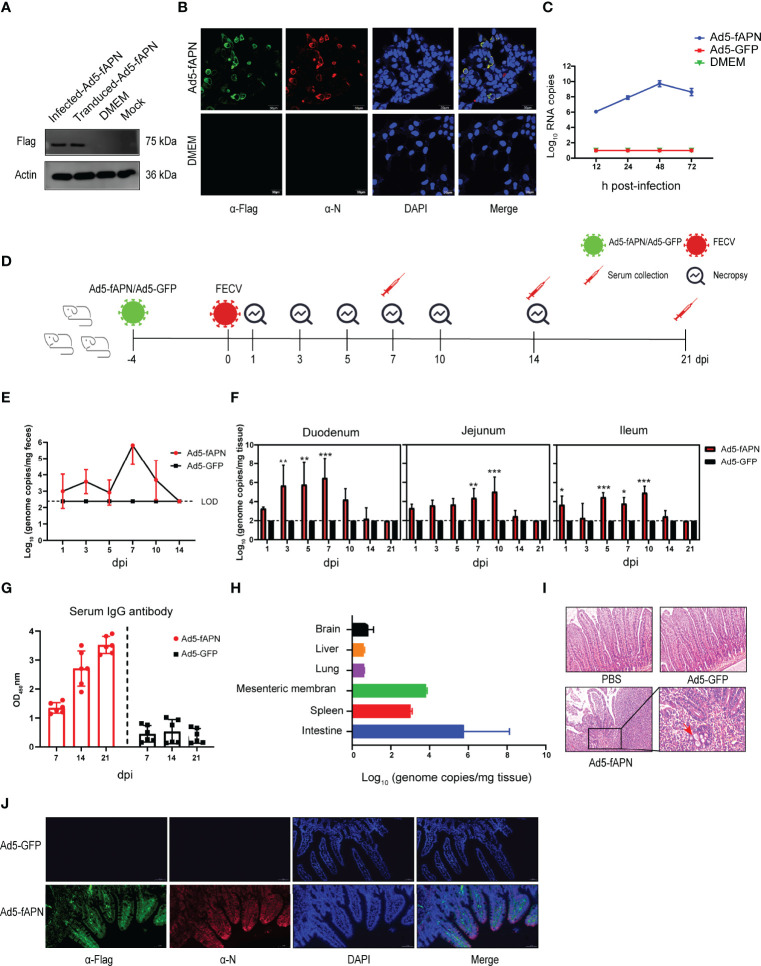
Generation of mice susceptible to FECV infection by transduction with fAPN. **(A)** Western blot of fAPN expression on Ad5-fAPN-transduced 293 cells. **(B)** Confocal image of FECV infection after Ad5-fAPN transduction. **(C)** Ad5-fAPN-transduced cells were infected with FECV at an MOI of 0.01 at 12, 24, 48, and 72 h post-transduction, and virus RNA titers were determined by qPCR. **(D)** Creation of mice susceptible to FECV infection. 2.5 × 10^6^ TCID_50_ of Ad5-fAPN/Ad5-GFP was injected intraperitoneally (i.p.) in 5-week-old mice, and 4 d later, 10^5^ TCID_50_ of FECV was i.p. injected. Feces and small intestinal tissue were collected at fixed time points to monitor FECV replication. **(E)** Viral RNA in the feces was determined by qPCR; n = 3 mice in each group at 1, 3, 5, 7, 10, and 14 dpi. **(F)** FECV RNA load in duodenum, jejunum, and ileum were determined at 1, 3, 5, 7, 10, 14, and 21 dpi by qPCR; n = 3 mice per group. **(G)** Anti-FECV IgG levels were determined by ELISA in sera from infected mice. Error bars in all samples indicate standard deviation, n = 3 mice per group at each time point. **(H)** FECV RNA load in the brain, liver, lung, mesenteric membrane, and spleen was determined at 5 dpi by qPCR; n = 3 mice per group. **(I)** Intestines were collected from mice at the indicated time points, fixed in zinc formalin, and embedded in paraffin. Sections were stained with hematoxylin/eosin and examined for microscopic lesions. **(J)** Double-labeling immunofluorescence cell staining analysis in the mouse intestinal tract. fAPN-Flag is labeled in green, FECV N protein is labeled in red, and nuclei are stained with DAPI (blue). *P<.05; **P<.01; ***P<.005

To generate mice that express the type II FECV receptor fAPN and are thus susceptible to FECV infection, we first developed a replication-deficient adenovirus carrying fAPN (Ad5-fAPN), with an adenovirus expressing GFP (Ad5-GFP) used as the control (**Figure 4A**). The vector system was first tested for its ability to make the FECV-refractory 293 cell line susceptible to FECV infection. FECV-positive cells were identified by IFA only in cells transduced with Ad5-fAPN (**Figure 4B**). To confirm cell susceptibility was due to fAPN expression, a one-step growth curve was performed; FECV replicated to high RNA titers only in Ad5-fAPN transduced cells (**Figure 4C**).

Next, an animal study was conducted to determine whether fAPN transduction could render mice susceptible to FECV infection (**Figure 4D**). As expected, in fAPN-transduced mice, the virus was shed in feces on the first day and peaked at 10^6^ genome copies/mg on the seventh day after infection (**Figure 4E**), whereas mice transduced with the GFP control had no detectable viral RNA load throughout the study. Viral genomic RNA was detected in the intestinal tract of fAPN-transduced mice (**Figure 4F**): The duodenum showed a high viral RNA load (10^6^ genome copies/mg) within 7 days after infection and gradually decreased after that, whereas the jejunum and ileum reached a maximum of 10^5^ genome copies/mg and had almost undetectable levels by 14 dpi. The mice had no significant changes in body weight throughout the experiment (data not shown). FECV-infected, Ad5-fAPN-transduced mice also had specific IgG antibodies in their sera, which reached the highest levels at least three weeks post-infection (**Figure 4G**). Furthermore, no viral RNAs were detected in other organs such as the brain, liver, or lungs at 5 dpi, though a small amount of viral replication (10^3^ to 10^4^ genome copies/mg) was detected in the mesentery and spleen (**Figure 4H**).

In order to observe possible microscopic pathology associated with FECV infection in Ad5-fAPN-transduced mice, intestinal sections of infected mice were analyzed by histopathology. Active FECV infection caused intestinal granuloma, focal necrosis with inflammatory cell infiltration, hyperemia of the villi tips, and atrophy of the villi (**Figure 4I**). Consistent with the histological lesions, immunofluorescence revealed robust viral N antigen and fAPN co-expression in the small intestines of mice transduced with Ad5-fAPN (**Figure 4J**). When FECV isolate 79-1683 was administered orally to specific pathogen-free kittens, mild enteritis similar to that of the mouse model was induced, with the virus occurring mainly in the small intestine and mesenteric lymph nodes (33). Based on the above results, we demonstrated the Ad5-fAPN-transduced mice to be an excellent model for surrogates of the type II FECV infection in their natural host.

### Oral Administration of rBS^CotB-HR2P^ Spores Effectively Immunize Susceptible Mice Against FECV Infection

**Figure 5:**
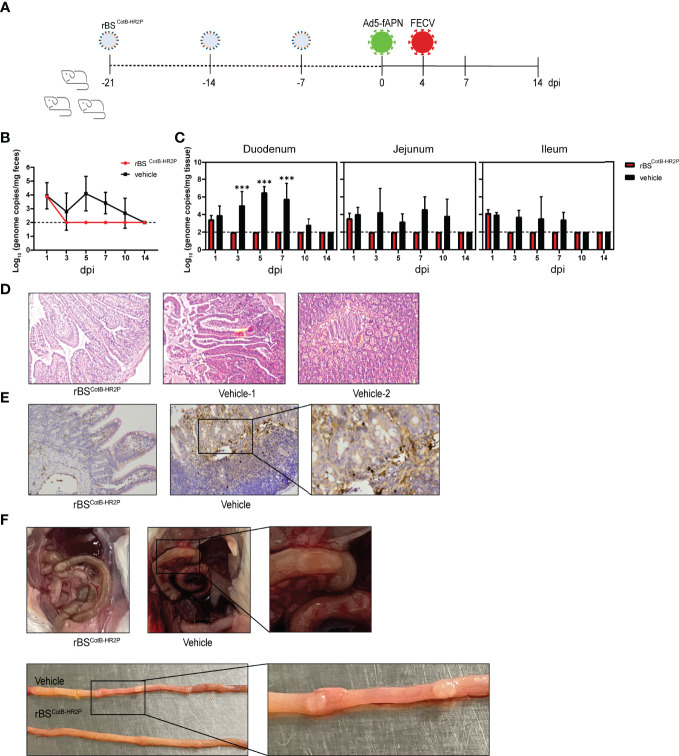
Immunization with rBS^CotB-HR2P^ protected mice against FECV infection. **(A)** Mice were inoculated with either rBS^CotB-HR2P^ or PBS (Ctrl) by gavage on days 1–3, 8–10, and 15–17. In order to make the mice susceptible to FECV infection, each was injected intraperitoneally (i.p.) with 2.5 × 10^6^ TCID_50_ Ad5-fAPN/Ad5-GFP, and 4 d later, each was i.p. challenged with 10^5^ TCID_50_ of FECV. **(B)** Viral RNA in feces was quantified by qPCR; n = 3 mouse in each group at 1, 3, 5, 7, 10, and 14 dpi. **(C)** FECV RNA load in the duodenum, jejunum, and ileum was determined at 1, 3, 5, 7, 10, and 14 dpi by qPCR; n = 3 mice per group. **(D)** Representative hematoxylin/eosin-stained section of mouse intestine. **(E)** IHC staining of the intestine for FECV N protein, showing viral replication. **(F)** Representative gross pathologic lesions found in the small intestine (intestinal granulomatous lesions in the duodenum). ***P<005

Our final step was the evaluation of the protective ability of the candidate FCoV vaccine in a novel mouse infection/challenge model. After three weeks of oral gavage with rBS^CotB-HR2P^ spores, mice were transduced with Ad5-fAPN by intraperitoneal injection (to make mice susceptible to FECV), and 4 days later, injected with 10^5^ TCID_50_ of FECV (**Figure 5A**). Viral shedding in feces was undetectable in immunized mice by 3 dpi, unlike naïve mice (**Figure 5B**). The virus was detected in the intestinal tissues of the groups at 1 dpi but became undetectable in the immunized group thereafter (**Figure 5C**). At 5 dpi, H&E staining showed that following FECV infection, non-immunized mice developed mild enteritis and some intestinal villi were necrotic and shed. However, there were no significant intestinal lesions in mice immunized with rBS^CotB-HR2P^ (**Figure 5D**). IHC showed that the infection spread widely in the cells of the small intestine in non-immunized mice. In contrast, the number of FECV-positive cells was much lower in the small intestines of the mice treated with rBS^CotB-HR2P^ (**Figure 5E**). Consistent with these findings, gross pathology was observed primarily in the intestine, with increased granulomatous lesions and abdominal bleeding in non-immunized mice (**Figure 5F**). The rBS^CotB-HR2P^ treatment reduced intestinal lesion severity greatly, and body weight was not affected by immunization status (data not shown).

## Discussion

Multiple CoV peptides derived from the S protein HR region are targets of viral entry/fusion inhibitors. Exogenous HR2P can compete with those on the virion, blocking the six-helix bundle formation and thus inhibiting viral fusion with the target cell (17, 18). We confirmed that recombinant HR2P effectively inhibited FECV infection and stimulated strongly neutralizing antibodies (**Figure 1**). Therefore, HR2-derived peptide is not only a potential antiviral drug for direct application to FCoV-related diseases but may also serve as a primary immunogen in protective vaccines. Generally, IM inoculation elicits a good immune response, but many adverse reactions have been reported after injection of peptides, such as enfuvirtide, which is used in HIV treatment (42, 43). The potential adverse effects of HR2P injection in cats are unknown; thus, finding a suitable vector for the delivery of the antigen is needed to address the FCoV infection. Therefore, this study developed a novel FCoV vaccine based on rBS^CotB-HR2P^ spores and evaluated its immunogenicity and protective ability against FECV in a novel mouse infection model. We clearly validated BSSD of the HR2P to be a safe, effective, and highly immunogenic option for vaccination of susceptible mice against FECV.

Since FCoV first invades the intestine, mucosal immunity is a key barrier that must be strengthened by any effective vaccine, and sIgA plays a major role in early infection (44). *B. subtilis* is well known for its generally-recognized-as-safe certification (45). It has been widely used in animal husbandry as a feed additive due to its probiotic effects and as a vaccine vector to target the gut (35, 46–49). *B. subtilis* spores have natural adjuvant activity, which can effectively increase T-cell reactions and promote IgA antibody production (27, 50). Spores can pass through the gastrointestinal barrier, germinating effectively in the gastrointestinal tract and growing and sporulating anaerobically. CotB encodes the spore capsid protein of *B. subtilis* (11), so the CotB-HR2P fusion is expressed only in large quantities when *B. subtilis* is in spore form. Of course, in the vegetative mass, there may be a small amount of CotB produced by leakage expression. We cannot rule out that HR2P expressed in the vegetative mass could also contribute to the observed immune response. Our results demonstrated that rBS^CotB-HR2P^ spores activate a strong immune response in the intestinal mucosa in addition to a broad general immune response and colonize the intestinal tract of the mice effectively, which likely improves the immune response and reduces the dosage requirement (**Figure 3**).

Adenovirus vectors have prominent advantages in transgenic expression and can sensitize mice to systemic infection (39). The E1/E3-deleted replication-deficient adenovirus vectors are the most extensively used vehicles for the delivery of therapeutic genes (51, 52). Here, we transduced mice with recombinant non-replicating adenovirus expressing the type II FECV receptor fAPN to establish a mouse model for FECV. This method has been successfully applied to rapidly generate mouse models for the zoonotic CoV pathogens MERS-CoV, SARS-CoV, and SARS-CoV-2, also by transduction of the respective entry receptors (37, 53, 54).

The establishment of a mouse infection model is an important step to further studying the pathogenesis of FCoV. Currently, the use of cats for FCoV research has certain limitations. Firstly, the supply of laboratory cats is very scarce, particularly in China. Secondly, there is no standardized breed of cats, which may lead to bias in experimental results. Third, animal welfare concerns for cats are an important issue to be considered in laboratory animal research (55). The use of adenovirus vectors to express the type II FCoV receptor fAPN in otherwise refractory mice was efficient and quick, permitting FECV infection in the intestinal tract. This infection resulted in mild disease (**Figures 4**, **5**) similar to the pathological phenotype of type II FECV 79-1683 infection in cats, including increased granulomatous lesions and abdominal bleeding (33). This mouse model can effectively replace experimental cats, and we expect it to play an important role in drug evaluation and antiviral therapy (11). However, a continuing limitation is the inability to study type I FCoVs due to their unknown receptors (9).

Using this FECV mouse model, we validated the immune effect of rBS^CotB-HR2P^ in triggering both anti-HR2P IgG and sIgA. The results also showed that mice immunized orally with rBS^CotB-HR2P^ provided protective immunity against FECVs (**Figure 5**). Taken together, we demonstrated that this BSSR technology-based rBS^CotB-HR2P^ is a promising oral vaccine candidate for the prevention and treatment of FECV infection. Additionally, due to the unique characteristics of *B. subtilis* spores (resistant to harsh conditions that can smoothly pass through the gastrointestinal barrier), rBS^CotB-HR2P^ may also serve as a probiotic feed additive in cat food to minimize the stress response of animals. Since the HR2 region is highly conserved among different FCoVs, we expect that rBS^CotB-HR2P^ has great potential in controlling other FCoV-related diseases. Further investigation of this novel approach and the protective effects against pathogenic FIPV in cats is ongoing.

## Data Availability Statement

The raw data supporting the conclusions of this article will be made available by the authors, without undue reservation.

## Ethics Statement

The animal study was reviewed and approved by Zhejiang University Experimental Animals Ethics Committee.

## Author Contributions

Y-WH, CC, and L-DX conceived and designed the study. CC, Y-LL, L-DX, and F-LL performed the experiments. CC, Y-LL, L-DX, and Y-WH analyzed the data. CC, L-DX, and Y-WH wrote the paper. All authors listed have made a substantial, direct, and intellectual contribution to the work and approved it for publication.

## Funding

This work was supported by the Laboratory of Lingnan Modern Agriculture Project (NG2022001), the Key Research and Development Program of Zhejiang province (2021C02049), and the Zhejiang Provincial Natural Science Foundation (LZ22C180002).

## Conflict of Interest

The authors declare that the research was conducted in the absence of any commercial or financial relationships that could be construed as a potential conflict of interest.

## Publisher’s Note

All claims expressed in this article are solely those of the authors and do not necessarily represent those of their affiliated organizations, or those of the publisher, the editors and the reviewers. Any product that may be evaluated in this article, or claim that may be made by its manufacturer, is not guaranteed or endorsed by the publisher.
